# Effectiveness analysis of arthroscopy-assisted medial retinaculum tightening suture with internal fixation for treating adolescent acute patellar dislocation with osteochondral fracture: A retrospective study

**DOI:** 10.1097/MD.0000000000047472

**Published:** 2026-01-30

**Authors:** Yanbo Wang, Yanlong Liu, Yuncong Ji, Dongqiang Yang, Biao Guo

**Affiliations:** a Department of Orthopaedics, Sports Medicine and Arthroscopy, Fuyang People's Hospital Affiliated to Anhui Medical University, Anhui, 236000, China; b Department of Orthopaedics, Sports Medicine and Arthroscopy, Fuyang People’s Hospital, Fuyang City, Anhui 236000, China.

**Keywords:** acute patellar dislocation, arthroscopic, chondral degeneration, osteochondral fracture, tightening suture

## Abstract

This study aims to investigate the therapeutic effectiveness of arthroscopically assisted medial retinaculum tightening suture, coupled with internal fixation, in managing acute patellar dislocation (APD) complicated by osteochondral fracture (OCF) in adolescent patients. We retrospectively analyzed the clinical data of 20 adolescents with APD with OCF who underwent arthroscopically assisted medial retinaculum tightening suture with internal fixation between April 2018 and April 2023. Eligible patients were younger than 20 years and had radiologically and arthroscopically confirmed osteochondral defects of the patella treated by fixation. Patients with previous patellar dislocation, ligament injuries, or preexisting patellofemoral osteoarthritis were excluded. The primary outcome was improvement in patellofemoral function, we evaluated the patient’s range of motion, clinical functional recovery, and pain alleviation by comparing the patellar tilt angle, lateral patellar displacement, Kujala Patellofemoral Joint score, Lysholm Knee score, International Knee Documentation Committee Motion Level score, and Visual Analogue Scale at preoperative, 3 months, and 1 year after surgery. The secondary outcome was to assess the thickness of the patellar cartilage in both knees using bilateral knee MRI scans and to evaluate early degenerative changes. For continuous variables that adhered to a normal distribution, either a *t* test or repeated-measures ANOVA was applied; Friedman test was used for continuous variables that did not conform to a normal distribution, with *P* < .05 considered statistically significant. Twenty adolescents were included, with a mean follow-up of 36.15 ± 12.42 months. Patellar tilt angle and lateral patellar displacement improved from 23.55 ± 6.73° and 21.39 ± 4.51 mm preoperatively to 11.23 ± 5.05° and 12.25 ± 4.55 mm at 3 months, and remained stable at 1 year (all *P* < .001 vs preoperative; *P* > .05 vs 3 months). At 3 months, Kujala, Lysholm, International Knee Documentation Committee, and Visual Analogue Scale scores were 64.80 ± 3.72, 67.60 ± 3.53, 82.70 ± 3.31, and 2.00 (1.00–3.00), all improved versus baseline (*P* < .001), with further improvement at 1 year (*P* < .001). No redislocation, subluxation, or significant patellar cartilage thinning versus the contralateral knee was observed on MRI. Arthroscopy-assisted medial retinaculum tightening suture with internal fixation provides reliable mid-term restoration of patellofemoral stability and function in adolescents with APD and OCF, without evidence of early patellar cartilage degeneration, and may represent a useful alternative option to medial patellofemoral ligament reconstruction in this population.

## 1. Introduction

Patellar dislocation predominantly occurs in adolescents and is more common in females,^[1]^ with acute traumatic incidents frequently resulting in damage to the medial retinaculum, which is critical to patellar stability.^[2]^ Such injuries may precipitate further dislocations. The incidence of acute patellar dislocation (APD) is approximately 23.2 per 100,000 individuals, soaring to approximately 147.7 per 100,000 among adolescents aged 14 to 18 years. This condition is often accompanied by hemarthrosis and cartilage damage,^[3,4]^ particularly during dislocation and realignment, where interactions between the medial patellar facet and the lateral femoral condyle may cause osteochondral fractures (OCFs) in these areas.^[5,6]^ The prevalence of these fractures ranges from 5% to 54%.^[7,8]^ If not adequately addressed, this condition can lead to early-onset degenerative joint disease in the patellofemoral joint, markedly impairing patient’s mobility and quality of life.

As the growth plates in adolescents have not yet been fused, inappropriate interventions can negatively impact the normal development of these plates and the overall recovery of the patient. Arthroscopy offers the advantages of minimal invasiveness, expedited recovery, and fewer complications. It provides a clear visual field, allowing for rapid verification of the size and location of OCFs, enhancing the precision of lesion identification and treatment efficacy. Numerous studies have corroborated the effectiveness of medial patellar femoral ligament (MPFL) reconstruction in treating patellar dislocation.^[9–11]^ However, for adolescents with open growth plates, MPFL reconstruction involving femoral drilling may damage these plates. An alternative method, arthroscopic tightening of the medial retinaculum, effectively reinstates normal anatomy and stability of the patellofemoral joint. Compared with MPFL reconstruction, this technique secures and repairs the patella without jeopardizing the growth plates, thus minimizing the likelihood of subsequent complications.

Therefore, the aim of this retrospective study was to evaluate the mid-term clinical and radiological outcomes of arthroscopy-assisted medial retinaculum tightening suture combined with internal fixation for treating adolescent patients with 1st-time APD and associated osteochondral fracture, and to explore whether this technique is associated with early degenerative changes in patellar cartilage.

## 2. Materials and methods

### 2.1. General information

We retrospectively analyzed the clinical data of adolescent patients treated at our hospital for APD accompanied by OCFs between April 2018 and April 2023. This was a single-center retrospective cohort study of consecutive patients who met the predefined eligibility criteria. The study was approved by the Institutional Review Board of our hospital ([2024]221), and written informed consent was obtained from all patients (and/or their legal guardians).

Inclusion criteria: under 20 years of age; a clear history of knee flexion and valgus sprain accompanied by sensations of patellar dislocation; patellar dislocation occurs for the first time; confirmed patellar dislocation and cartilage fractures through CT or MRI; arthroscopic evidence of cartilaginous defects on the patellar articular surface, with matching free cartilaginous fragments in the joint cavity.

Exclusion criteria: absence of significant cartilage damage on imaging; existing patellofemoral osteoarthritis; previous history of patellar dislocation; concurrent anterior or posterior cruciate ligament injuries; potential infectious risks at the surgical site; psychiatric conditions precluding surgical tolerance or postoperative rehabilitation compliance.

### 2.2. Surgery technique

All procedures were performed by the same senior sports medicine physician. Following the successful administration of anesthesia, the patient was positioned supine with the lower limb flexed at a 90° angle for knee flexion, routinely disinfected, draped, and connected to the arthroscopic equipment. An inflatable tourniquet on the lower limb was inflated at 0.070 mpa.

At the level of the knee joint gap, 2 incisions measuring 0.8 cm each were made on either side of the patellar ligament, spaced 0.5 cm apart, in an inverted “eight” shape to facilitate medial incision extension directly into the joint cavity. The arthroscope was introduced into the knee joint cavity through the lateral portal, and a systematic examination of the cavity was performed to ascertain the location of any OCFs, confirming the presence of that cartilage damage on the patellofemoral subtalar articular surface while identifying free cartilage bone masses within the articular cavity to evaluate their quantity, size, shape, and integrity. The position, extent, size, and depth of the patellofemoral bone defect were subsequently confirmed in relation to the compatibility with free bone masses. Blood accumulation within the joint was cleared as necessary. Additionally, an appropriate extension of the medial approach facilitated the removal of chondral fracture fragments.

An incision was made along a medial arc line adjacent to the patella measuring approximately 6 to 8 cm was made; which involved cutting through supportive bands and a joint capsule at the inner edge of the patella. Two 2.0-gram pins were drilled medially into the patella while assisting external rotation at approximately 90° for clear visualization of fracture trauma. Cartilage fragments were reset and secured using 3 to 5 cartilage nails such that their ends aligned flush with the cartilage surface. Examination confirmed proper resetting of the fractures and satisfactory positioning of the internal fixation.

The medial collateral ligament was freed from adhesions and reinforced using high-strength wire with a generous margin of at least 10 to 15 mm; it was tightened and reattached to provide support by suturing proximally at the junction with the medial femoral muscle and distally at the lower edge level of the patella with stitch spacing set at 10 mm (typically requiring 6 stitches for closure) overlapping them before tying knots for fixation. Notably, significant reinforcement and tightening of the medial collateral ligament were observed alongside increased tension in extrapolation compared with preoperative assessments.

**Figure 1.**
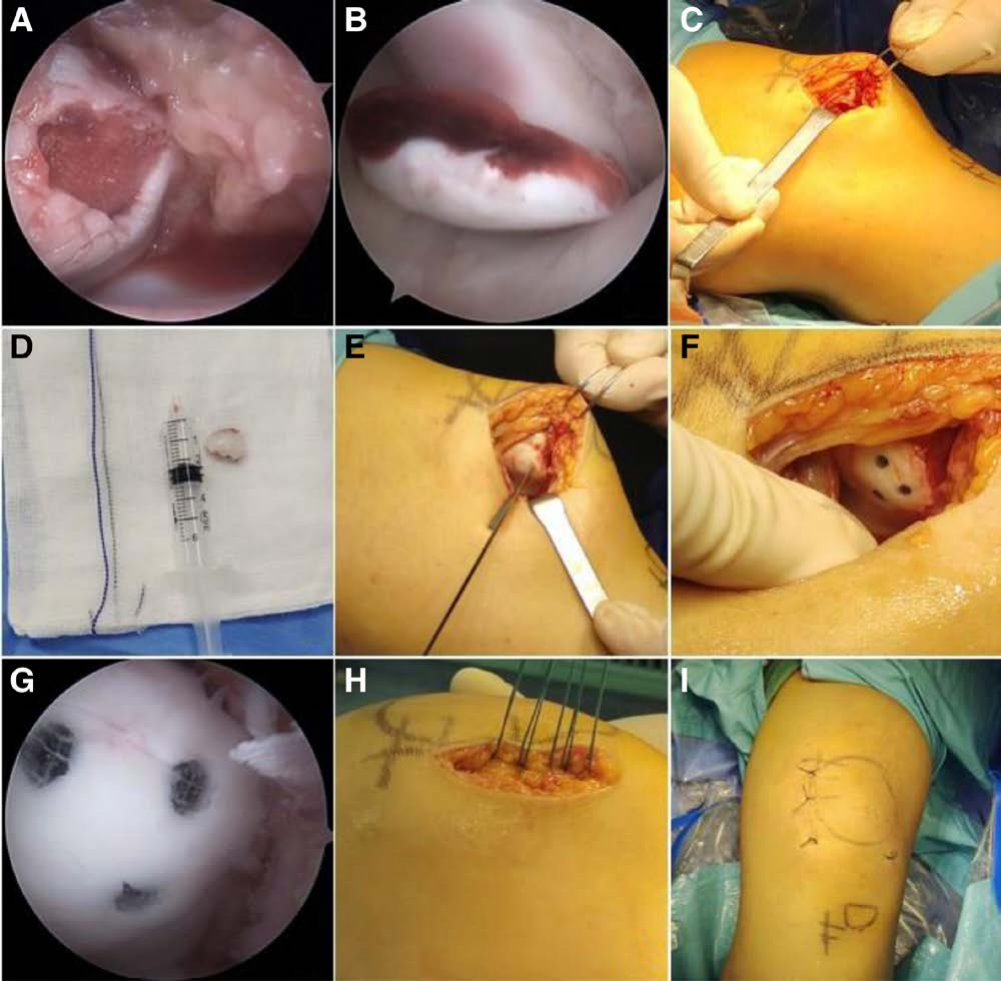
(A) Arthroscopic exploration of the knee joint cavity and visualization of the patellar defect site. (B) Arthroscopic examination of the free OCF. (C) Incision to reveal the patellar defect. (D) Removal of OCF with confirmation of their dimensions. (E) Reduction of the OCF followed by temporary fixation. (F) Stabilization of the OCF using cartilage nailing. (G) The arthroscopic examination reveals that the OCF is securely reset. (H) Tightening suture accompanied by medial retinaculum. (I) Skin incision and suturing without resulting in a scar. OCF = osteochondral fracture.

Upon lens observation during flexion and extension ranging from 0° to 90°, satisfactory alignment between patellofemoral joints throughout movement was noted alongside an acceptable trajectory during these motions. The wound was thoroughly irrigated, followed by hemostasis; skin incisions were closed without scarring before applying an elastic bandage pressure dressing after releasing tourniquet support around knee stabilization (Fig. 1).

### 2.3. Postoperative treatment

All patients were fitted with knee chucks for support following surgery and engaged in rehabilitation training under the expert guidance of professional rehabilitation trainers. On the second postoperative day, they commenced ankle pump exercises and quadriceps isometric contraction training. Upon achieving full activation of the medial femoral muscle, they progressed to straight leg raises and lateral leg raises, as well as patellar mobilizations: pushing the patella upward, downward, and inward. They also initiated flexion and extension mobility training within a pain-free range, achieving a knee joint range of motion of 90° by 2 weeks post-surgery and 120° by 6 weeks. Furthermore, on the 2nd day after surgery, progressive weight-bearing exercises could be performed with the brace providing protection; the brace was subsequently removed after 6 weeks to facilitate the restoration of normal gait.

### 2.4. Evaluation metrics

Patellar tilt angle (PTA) and lateral patellar displacement were assessed using CT or MRI of the knee joint both preoperatively and postoperatively. A comparative analysis of PTA and lateral patellar displacement was conducted among patients before surgery, 3 months after surgery, and 1 year after surgery.

The preoperative, 3-month postoperative, and 1-year postoperative scores of the patients, along with the International Knee Documentation Committee (IKDC) scores (0–100), Lysholm scores (0–100), Kujala scores (0–100), and Visual Analog Scale (VAS) scores were meticulously compared to assess the restoration of knee function.

**Figure 2.**
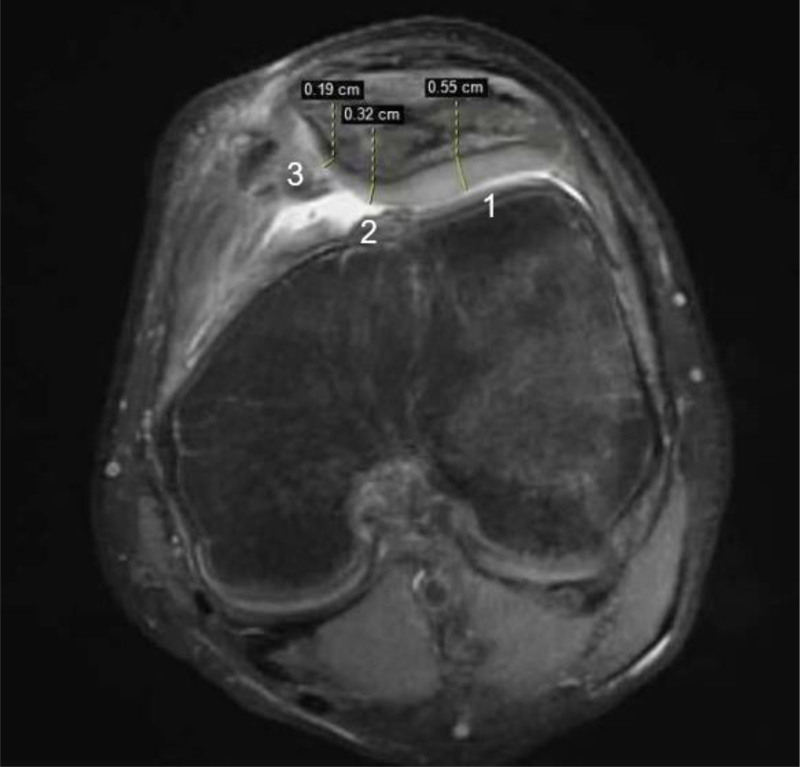
MRI cross-section with reference points.

During the final follow-up, MRI examinations of both knees were repeated, allowing for a comparison of the patellar cartilage thickness between the affected knee joint and its contralateral counterpart to evaluate early degenerative changes in the cartilage of the affected knee joint. The measurement of the patellar cartilage was conducted using 3 reference points in the cross-section: point 1 – cartilage on the lateral surface; point 2 – cartilage at the ridge of the patellar articular surface; and point 3 – cartilage on the medial articular surface^[12]^ (Fig. 2).

### 2.5. Statistical analysis

The SPSS 26.0 statistical software was employed to analyze the data, utilizing the Shapiro–Wilk method to assess the normality of the dataset. For continuous variables that adhered to a normal distribution, either a *t* test or repeated-measures ANOVA was applied, with results presented as mean ± standard deviation ($\bar{x}\pm s$). Conversely, Friedman test was used for continuous variables that did not conform to a normal distribution, and the measurement data were expressed as median and interquartile range [M(P25, P75)]. A *P*-value <.05 was deemed indicative of a statistically significant difference.

## 3. Results

**Figure 3.**
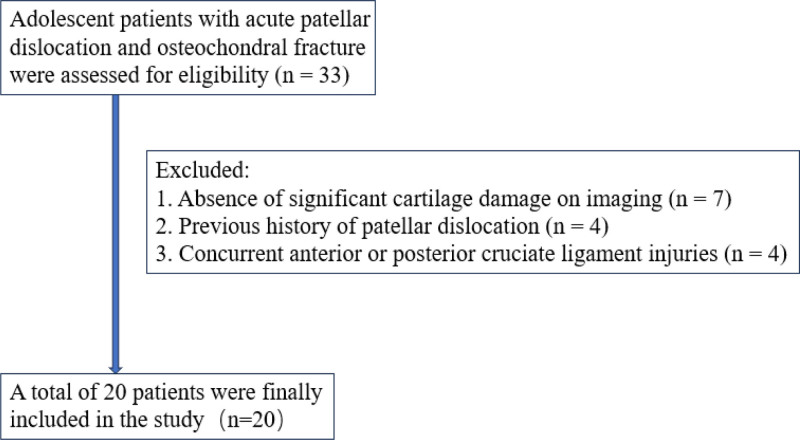
Flowchart showing inclusion and exclusion of patients in the study.

A total of 20 adolescent patients fulfilled the inclusion criteria during the study period. There were 9 males and 11 females with a mean age of (15.75 ± 1.97) years. The inclusion and exclusion process is illustrated in Figure 3. Nine instances involved the left knee and 11 involved the right knee. All cases were acute injuries: 15 were attributed to daily life incidents and 5 to sports-related activities. Patient heights ranged from 1.57 to 1.87 m, averaging (1.71 ± 0.84) m, and weights spanned from 44.0 to 120.0 kg, with an average of (67.75 ± 17.19) kg. Preoperative body mass index varied from 15.73 to 34.32 kg/m^2^, averaging (23.00 ± 4.21) kg/m^2^. All patients received comprehensive follow-up, with an average duration of (36.15 ± 12.42) months (range, 12–50 months).

### 3.1. Clinical therapeutic effectiveness

#### 3.1.1. MRI assessment

The PTA decreased significantly from a preoperative average of (23.55 ± 6.73)° to (11.23 ± 5.05)° at 3 months postoperation, and the lateral displacement of the patella reduced notably from a preoperative measurement of (21.39 ± 4.51) mm to (12.25 ± 4.55) mm at 3 months postoperation, both differences being statistically significant. There were no significant changes in PTA and lateral displacement rate between 3 months and 1 year postoperation (Table 1).

**Table 1:** Comparison of patients’ preoperative and postoperative patellar position results (n = 20, $\bar{x}\pm s$).

Time	PTA (°)	LPD (mm)
Preoperative	23.55 ± 6.73	21.39 ± 4.51
Three months after surgery	11.23 ± 5.05	12.25 ± 4.55
One year after surgery	11.80 ± 3.90	11.79 ± 4.31
*F*-value	104.99	99.88
*P*-value	<.001	<.001

LPD = lateral patellar displacement, PTA = patellar tilt angle.

#### 3.1.2. Knee functionality score

The Kujala score for patellofemoral joint assessment improved markedly from a preoperative score of (43.20 ± 3.52) to (64.80 ± 3.72) at 3 months postoperation, with the differences being statistically significant (*P* < .001). Similarly, the Lysholm score for knee function enhanced significantly from a preoperative score of (33.90 ± 3.37) to (67.60 ± 3.53) at 3 months postoperation, as did the IKDC sports activity level rating which escalated impressively from a preoperative tally of (51.03 ± 3.62) to an encouraging (82.70 ± 3.31) at 3 months postoperation, with the differences being statistically significant (*P* < .001). All assessments (the Kujala Patellofemoral Joint score, Lysholm Knee score, and IKDC Motion Level score) showed further improvement 1 year after surgery compared with their values at 3 months postoperation, with all differences being statistically significant (Table 2).

**Table 2:** Comparison of preoperative and postoperative knee joint functional scores in patients [n = 20, $\bar{x}\pm s$ or M(P25, P75)].

Time	Kujala score	Lysholm score	IKDC score	VAS score
Preoperative	43.20 ± 3.52	33.90 ± 3.37	51.03 ± 3.62	5.00 (4.00–7.00)
Three months after surgery	64.80 ± 3.72	67.60 ± 3.53	82.70 ± 3.31	2.00 (1.00–3.00)
One year after surgery	91.70 ± 3.03	92.95 ± 2.19	94.31 ± 2.52	0.00 (0.00–1.00)
*F*-value	1444.954	2354.692	1077.903	
*P*-value	<.001	<.001	<.001	<.001

#### 3.1.3. VAS score

Preoperatively, patient VAS scores averaged at 5 points on a scale of 5.00 (4.00–7.00) but improved remarkably to 2.00 (1.00–3.00) scores after surgery and continued improving until reaching 0.00 (0.00–1.00) points by 1-year follow-up: there was a statistically significant difference by pairwise comparisons (*P* < .001) (Table 2).

### 3.2. Evaluation of early degenerative changes

MRI scans of the patellar joint cartilage revealed that compared with the healthy contralateral knee, there was no noteworthy thinning across any reference points on the patella (Table 3).

**Table 3:** Comparison of bilateral cartilage thickness at the final follow-up visit in patients (n = 20, $\bar{x}\pm s$).

	Medial articular surface	The ridge of the patellar articular surface	Lateral articular surface
Affected side	3.19 ± 0.47	3.80 ± 0.60	4.54 ± 0.85
Healthy side	3.22 ± 0.50	3.83 ± 0.67	4.51 ± 0.82
*T*-value	-0.543	-0.267	0.313
*P*-value	.594	.792	.757

### 3.3. Typical case

**Figure 4.**
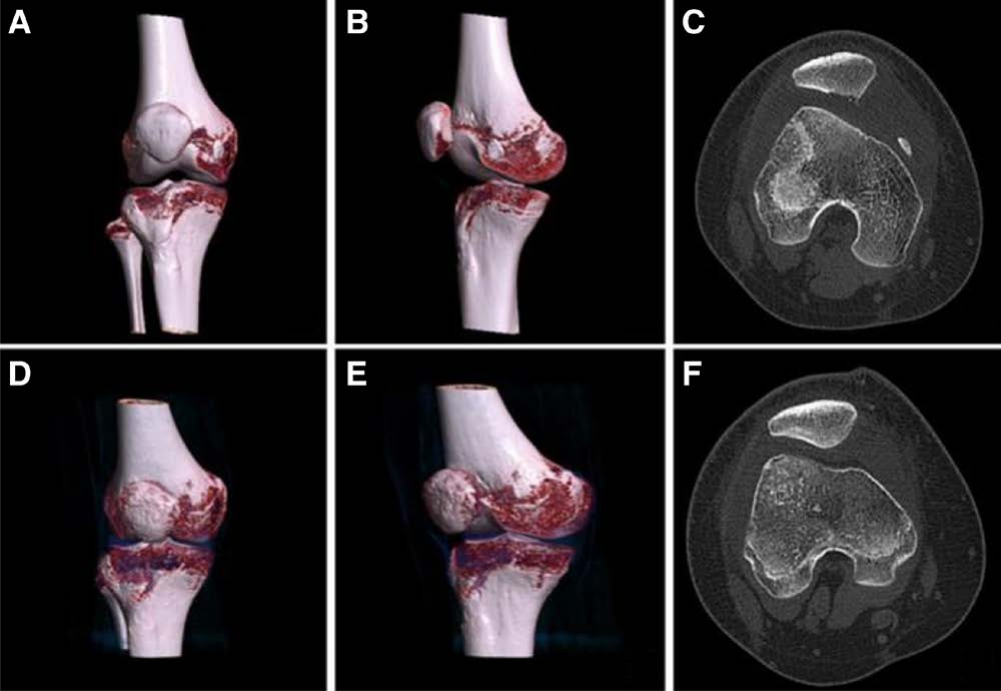
(A–C) Preoperative 3-dimensional CT reconstruction of the right knee. (D–F) Three-dimensional CT reconstruction of the right knee at 3 months after surgery.

The patient, a 15-year-old female, experienced pain and restricted movement in her right knee following a twisting injury sustained while walking. Preoperative 3-dimensional CT reconstruction of the right knee indicated a bone defect at the posterior edge of the patella and loose bone fragments at the anterior edge of the medial femoral condyle. Three months post-surgery, the patient’s knee exhibited normal flexion and extension movements (Fig. 4).

## 4. Discussion

A crucial component of the knee joint is compromised when it deviates from its normal position, leading to pain, a sense of instability, and functional impairment. The stability of the patella relies on a myriad of factors, such as the interaction of soft tissues (quadriceps muscle, patellofemoral and patella-tibial ligaments, and patella retinaculum) and bone (trochlear groove depth, engagement of the patella and groove during flexion, and height of the patella-alta, Baja).^[13]^ The medial retinaculum plays a vital role in maintaining the inward stability of the patella, preventing lateral dislocation during knee flexion.^[14]^ Medial retinaculum tear or injury compromises the ability of the patella to maintain its normal anatomical position, significantly increasing the risk of recurrent dislocations. This study utilized arthroscopy-assisted medial retinaculum tightening suture with internal fixation for treating adolescent APD with OCF. The results demonstrated that this surgical approach effectively alleviates pain, improves function, and achieves favorable therapeutic outcomes.

Advantages of arthroscopically assisted medial retinaculum tightened with internal fixation in treating adolescent APD with OCF: significant postoperative pain relief. Patients with APD often experience pain and functional impairment due to medial retinaculum tears, soft tissue injuries, and concurrent OCFs. These injuries prevent the patella from maintaining its normal anatomical position. By performing medial retinaculum tightening sutures and fixing osteochondral fragments, the anatomical structure and mechanical function of the knee joint are restored, effectively reducing patellar instability and alleviating knee pain. In this study, patients showed significant improvement in VAS scores at both 3 months and 1 year postoperatively compared to preoperative levels. Currently, there are 2 main techniques for medial retinaculum tightening: radiofrequency-assisted tightening and suture tightening. Both approaches yield reliable clinical outcomes.^[15–17]^ This study employed the traditional suture tightening technique, wherein overlapping and pleating of the medial retinaculum were followed by suturing to maintain the patella in its appropriate anatomical position. This approach achieved favorable clinical results while minimizing trauma to the medial retinaculum. Furthermore, it avoids inflammatory reactions associated with thermal damage from radiofrequency devices, contributing to early postoperative pain relief. Significant improvement in knee joint function. Postoperative Lysholm scores, Kujala scores, and IKDC scores were all significantly higher than preoperative values in this study. Zhao et al^[15]^ conducted a 14.6-month follow-up on 24 adolescent patients with APD and found that postoperative functional scores were significantly improved compared to preoperative levels, with scores at 12 months surpassing those at 3 months. These findings align with the clinical outcomes observed in this study. Low incidence of complications. No complications occurred among the patients included in this study postoperatively. Previous research has shown that MPFL reconstruction achieves a success rate of 89% to 100% in preventing recurrent patellar dislocation,^[18]^ making it widely regarded as the gold standard treatment for patellar instability. However, MPFL reconstruction is not ideal for adolescent patients with open growth plates due to its potential risk of damaging the femoral physis at its attachment site,^[19]^ which may affect skeletal development. Additionally, MPFL reconstruction typically involves harvesting autologous tendons such as quadriceps tendon or semitendinosus tendon as grafts, which can lead to significant donor site morbidity and a high incidence of complications.^[20–22]^ In contrast, medial retinaculum tightening does not require bone tunnel preparation, thereby avoiding damage to open growth plates in skeletally immature patients. It is less invasive and easier to perform compared to MPFL reconstruction. Previtali et al^[23]^ compared MPFL reconstruction with medial retinaculum tightening suture techniques and retinacular repair procedures and found no significant differences in clinical outcomes between these methods. Similarly, Jin Jiang et al^[24]^ demonstrated that medial retinaculum tightening is equally effective as MPFL reconstruction or repair procedures in improving functional outcomes.

Limitations of this approach: longer operative time. Combining these 2 surgical techniques requires multiple procedures within a single operation, including arthroscopic exploration, osteochondral fragment fixation, and medial retinaculum tightening sutures. This increases technical demands on surgeons while extending operative time. Higher costs. Compared to traditional open surgery, arthroscopic procedures involve more sophisticated equipment and consumables, resulting in higher overall medical expenses and increased financial burden on patients.

Some scholars advocate simultaneous release to reduce the lateral pull of the lateral muscles on the patella, thereby increasing the medial movement of the patella and reducing the lateral tilt. However, studies have suggested that lateral support strap release should not be performed without specific physical evidence of lateral tissue tension.^[25]^ Song, GY et al^[26]^ recommended cautious selection of lateral support strap release in treating patients with patellar dislocation, especially when preoperative confirmation of lateral support strap tension is lacking. Excessive release and inappropriate indications for release surgery are the 2 factors that can lead to iatrogenic instability of the patella. In our study, the patients did not exhibit combined lateral support strap tension; hence lateral release was not required, as excessive loosening could lead to decreased lateral stability of the patella, thereby increasing the risk of iatrogenic medial dislocation.

The patients included in this study all had cartilage fractures and their OCFs were fixed with bioabsorbable nails while undergoing medial retinaculum tightening sutures. In our study, the patients had sustained cartilage fractures, and during medial retinaculum tightening, biodegradable screws were used to secure OCFs. At the final follow-up, the OCFs had healed well, with MRI showing successful integration of bone and cartilage fragments and satisfactory functional recovery. However, instability of the patella, cartilage fractures, and related surgical interventions might accelerate the overall degeneration of the joint cartilage, not just within the patellofemoral joint. Consequently, we focused on whether there were any early degenerative changes postoperatively.

Postoperative follow-up revealed no significant difference in cartilage thickness at any patellar reference point compared to the healthy side, suggesting that medial retinaculum tightening suture could restore the patella to its correct anatomical position, thereby reducing uneven stress, wear, and cartilage degeneration. This treatment is both effective and safe for patients with APD, without increasing the risk of cartilage degeneration. Although there is no specific research on degenerative changes in cartilage following medial retinaculum tightening sutures for APD, existing studies on medial patellofemoral ligament reconstruction provide some insights. Nomura E et al^[27]^ showed that this surgery effectively prevented recurrent dislocations in patients experiencing no or minor progression of knee arthritis. After a 12-year follow-up of 22 patients undergoing medial patellofemoral ligament reconstruction, only 2 joints showed progression of osteoarthritis at the final follow-up. Another study on children with patellar instability revealed that, after a 12-year follow-up, the superficial quality of the cartilage was good, with no significant difference compared to asymptomatic knees. However, further follow-up is required for the deeper cartilage layers.^[28]^ This suggests that patellar stabilization surgeries may offer some protection against cartilage degeneration by enhancing the support and stability of medial structures, thus reducing the occurrence and progression of cartilage degeneration following patellar dislocation and injury. Therefore, we speculate that tightening the suture of the medial retinaculum after APD may have a similar effect.

This study had relatively small sample sizes. A post hoc power analysis was performed using 3.1.9.7 statistical software for the 2-tailed paired-samples *t* test comparing preoperative and postoperative IKDC scores. Based on the observed effect size, a sample size of 20 patients, and a significance level of α = 0.05, the achieved statistical power (1 − β) exceeded 0.99. Therefore, although our cohort was relatively small, the study’s statistical power can be considered sufficient.

In addition, several methodological limitations of the present study should be acknowledged. First, this was a retrospective, single-center analysis with a relatively small sample size, which inevitably limits the statistical power and generalizability of the findings. Second, we did not include a control group treated with conventional non-operative management or alternative surgical procedures; therefore, direct comparisons between treatment strategies cannot be made, and selection bias cannot be excluded. Third, because of the limited sample size and retrospective design, we were unable to perform comprehensive multivariable modeling to adjust for potential confounders, including age, sex, body mass index, injury mechanism, lesion location and size, baseline anatomical risk factors, and adherence to postoperative rehabilitation. As a result, unmeasured or residual confounding may have influenced the observed associations between the surgical technique and clinical or radiological outcomes. Larger, multicenter prospective studies or randomized controlled trials with appropriate control groups and multivariable analyses are warranted to confirm these results and to define the comparative effectiveness of this approach more robustly.

## 5. Conclusion

In summary, our results suggest that arthroscopy-assisted medial retinaculum tightening suture with internal fixation of cartilage is a safe and effective treatment for treating adolescent APD with OCF, providing stable anatomical reduction, satisfactory mid- to short-term radiological and clinical functional scores, and good cartilage healing at the final follow-up, with no cases of redislocation. Furthermore, MRI examinations of any patient did not reveal any more significant degenerative changes compared to the healthy side, indicating that our surgical technique might effectively prevent or slow the progression of cartilage degeneration following patellar dislocation.

## Author contributions

**Conceptualization:** Yanbo Wang, Yanlong Liu.

**Data curation:** Yuncong Ji.

**Formal analysis:** Biao Guo.

**Investigation:** Yanbo Wang, Yanlong Liu, Yuncong Ji.

**Methodology:** Dongqiang Yang, Biao Guo.

**Resources:** Yanbo Wang, Yanlong Liu.

**Supervision:** Dongqiang Yang, Biao Guo.

**Validation:** Yanbo Wang, Yanlong Liu.

**Visualization:** Yanbo Wang, Yanlong Liu.

**Writing – original draft:** Yanbo Wang, Yanlong Liu.

**Writing – review & editing:** Yanbo Wang, Yanlong Liu, Biao Guo.
